# Supplementary material for: Characteristics and outcomes of a cohort of COVID-19 patients in the Province of Reggio Emilia, Italy
Source: PLoS One. 2020 Aug 27;15(8):e0238281. doi: 10.1371/journal.pone.0238281 (PMC7451640; doi:10.1371/journal.pone.0238281)
Supplement: S1 File — (DOC) [file pone.0238281.s001.doc]

| **ICD9-CM codes from hospital records and drug class ATC codes from outpatient pharmacy data for the identification of single comorbidities.** |
| --- |
| COPD (ICD9-CM 490, 491, 492, 493.22, 494, 496) or (ATC class R03BB) |
| Coronary heart disease (ICD9-CM 410, 411, 412, 413, 414) |
| Dementia (ICD9-CM 290, 2941, 3312) |
| Diabetes (ICD9-CM 250) or (ATC class A10) |
| Chronic kidney disease (ICD9-CM 585) |
| Cancers (ICD9-CM from 140 to 239) |
| Hypertension (ICD9-CM 401, 402, 403, 404, 405) |
| Obesity (ICD9-CM 278.0) |
| Heart failure (ICD9-CM 398.91, 402.01, 402.11, 402.91, 404.01, 404.03, 404.11, 404.13, 404.91, 404.93, 425, 428) |
| Arrhythmia (ICD9-CM 426.0, 426.13, 426.7, 426.9, 426.10, 426.12, 427, 785.0, 996.01, 996.04, V45.0, V53.3) |
| Dyslipidaemia (ICD9-CM 272) |
| Vascular disease (ICD9-CM 440, 441, 442, 443, 444, 445, 446, 447, 448, 557) |
