# Supplementary material for: Characteristics and outcomes of a cohort of COVID-19 patients in the Province of Reggio Emilia, Italy
Source: PLoS One. 2020 Aug 27;15(8):e0238281. doi: 10.1371/journal.pone.0238281 (PMC7451640; doi:10.1371/journal.pone.0238281)
Supplement: Attachment — Submitted filename: Response to Reviewers.docx [file pone.0238281.s002.docx]

1. Please ensure that your manuscript meets PLOS ONE's style requirements, including those for file naming. The PLOS ONE style templates can be found at

<https://journals.plos.org/plosone/s/file?id=wjVg/PLOSOne_formatting_sample_main_body.pdf> and

<https://journals.plos.org/plosone/s/file?id=ba62/PLOSOne_formatting_sample_title_authors_affiliations.pdf>

2. We note that you have included the phrase “data not shown” in your manuscript. Unfortunately, this does not meet our data sharing requirements. PLOS does not permit references to inaccessible data. We require that authors provide all relevant data within the paper, Supporting Information files, or in an acceptable, public repository. Please add a citation to support this phrase or upload the data that corresponds with these findings to a stable repository (such as Figshare or Dryad) and provide and URLs, DOIs, or accession numbers that may be used to access these data. Or, if the data are not a core part of the research being presented in your study, we ask that you remove the phrase that refers to these data.

RE: The differences in age and sex distribution of cases over the weeks of the epidemic are reported in Table 2. Regarding the proportion of people without hospitalizations reporting comorbidities, the data are reported in the text only and not in a table, so it is not true that “data [were] not shown”. Indeed, there was no reason to say, “data not shown”, sorry.

Funding: we previously stated that the study did not receive any external funding but in July 2020 we were notified that a proposal for a grant submitted by our group to the Italian Ministry of Health on this topic has been accepted (grant number COVID-2020-12371808). This grant will cover the publication costs and partially the cost sustained for collecting data. We added this in the Funding section.


[Note: HTML markup is below. Please do not edit.]

Reviewers' comments:

Reviewer's Responses to Questions

**Comments to the Author**

1. Is the manuscript technically sound, and do the data support the conclusions?

The manuscript must describe a technically sound piece of scientific research with data that supports the conclusions. Experiments must have been conducted rigorously, with appropriate controls, replication, and sample sizes. The conclusions must be drawn appropriately based on the data presented.

Reviewer #1: Yes

Reviewer #2: Yes

2. Has the statistical analysis been performed appropriately and rigorously?

Reviewer #1: Yes

Reviewer #2: I Don't Know

3. Have the authors made all data underlying the findings in their manuscript fully available?

The [PLOS Data policy](http://www.plosone.org/static/policies.action#sharing) requires authors to make all data underlying the findings described in their manuscript fully available without restriction, with rare exception (please refer to the Data Availability Statement in the manuscript PDF file). The data should be provided as part of the manuscript or its supporting information, or deposited to a public repository. For example, in addition to summary statistics, the data points behind means, medians and variance measures should be available. If there are restrictions on publicly sharing data—e.g. participant privacy or use of data from a third party—those must be specified.

Reviewer #1: Yes

Reviewer #2: Yes

4. Is the manuscript presented in an intelligible fashion and written in standard English?

PLOS ONE does not copyedit accepted manuscripts, so the language in submitted articles must be clear, correct, and unambiguous. Any typographical or grammatical errors should be corrected at revision, so please note any specific errors here.

Reviewer #1: Yes

Reviewer #2: Yes

5. Review Comments to the Author

Please use the space provided to explain your answers to the questions above. You may also include additional comments for the author, including concerns about dual publication, research ethics, or publication ethics. (Please upload your review as an attachment if it exceeds 20,000 characters)

Reviewer #1: In this Ms., Paolo Giorgi Rossi et al., reported a cohort of SARS-CoV-2 patients in Reggio Emilia. A total of 2653 symptomatic patients were included. They found that patients with heart failure, arrhythmia, dementia, ischemic heart disease, diabetes, and hypertensions show higher risk of hospitalization and of death. ACE inhibitors has no effect on risk of death. This Ms. was not well written. The novel findings in current study should be highlighted. The comparison with previous studies and corresponding interpretations should be more intensive.

RE: We have revised the Discussion and updated the references. We have focused on methodological issues that could explain the heterogeneity in results found in different studies.

Reviewer #2: The manuscript presents data on COVID 19 from patients in the Reggio Emilia Province. Data from symptomatic SARS COV 2 positive patients are set in relation with the routine database of the local health authority. Compared to other data, e.g. from china, Case fatality reported in this study is comparatively high. The manuscript is straight forward and well written. I therefore have only few comments:

RE: We thank the reviewer for his encouraging comments.

1. A major restriction of the study is, that mainly (this changed during the period) symptomatic patients were tested and only symptomatic Sars Cov 2 patients were included in the study. It is therefore impossible to deduct the “prevalence of infection” (e.g. abstract p.2 l.23, discussion p13 l 216) as only the prevalence of symptomatic infection is assessed. This must be changed throughout the manuscript. As a consequence, the data in table 1 (which is not Sars Cov 2 prevalence but prevalence of symptomatic COVID-19) has to be interpreted with caution. The prevalence is 10x higher in >81yrs males compared to <51ys. Is this because risk of infection is higher in elderly? Or just the rate of symptomatic patients in elderly? Or a higher rate of testing? This issue needs more attention in the manuscript.

RE: We agree with the reviewer that this is a limitation of our study; we cannot determine whether differences in disease occurrence were due to differences in the access to testing, to differences in the probability of infection, or to differences in the probability of having symptoms, once infection occurred. We are presenting data on cumulative incidence of COVID-19 diagnosed and confirmed through RT-PCR SARS-CoV-2 test according to the case definition adopted by the Italian Ministry of Health. To be consistent, we have substituted SARS-CoV-2 with COVID-19 in the manuscript where appropriate.

We have added a better description of how the swabs were taken, including a column in Table 1 reporting the number of swabs taken, by sex and age, and in Table 2 by epidemic period. This information helps when interpreting differences by age, showing that older people, at least in this phase of the epidemic, were probably tested when symptoms were more predictive of COVID-19 than were younger patients. This information highlights the population-based nature of the study. Furthermore, we have added a sentence in the limitations section of the discussion.

2. Linked with 1, it is not clearly stated how “symptomatic” was defined and how the physicians decided who was being tested (as only epidemiological data is used). If the authors could give more information here, this would certainly strengthen the manuscript.

RE: We reported in the text the exact list of symptoms adopted by the Italian Ministry of Health to guide field investigations during the epidemic. We have added a paragraph explaining how people had access to the test, which is a more context-specific information.

3. Prevalence of comorbidities is deducted from data on previous hospitalizations (routine database). As far as I understand, someone who was never hospitalized during the past 10 years and has e.g. hypertension or COPD managed in outpatient treatment would not be counted as a patient with comorbidity. This weakness is already discussed on p14 l227ff. In my view, at least for SARS positive patients – and for those the increased risk for hospitalization and death connected with pre-conditions was mainly calculated – pre-conditions should have been assessed during the “epidemiological interviews” (p6 l90) or certainly during hospitalization for COVID and should have been included in the “special database” of the cohort. Has this been done? If not it has to be clearly stated.

RE: During the epidemiological interviews, collection of comorbidities was not structured; this information may be present in a free text field used to report special situations, but reporting was surely not systematic. Further, while comorbidities are routinely collected during emergency room visits, but without using a structured form, reporting is once again not systematic. Finally, during hospitalization, the anamnesis is more systematically collected, but as the structured medical records are ward-specific, reporting is not uniform anyway. What we noted is that the probability of reporting comorbidity increased with the severity of disease and with the intensity of the care setting. The result is that the measure of comorbidities as a prognostic factor can be done only if we use a source of information that is independent of and possibly acquired before the COVID-19 diagnosis.

6. PLOS authors have the option to publish the peer review history of their article ([what does this mean?](https://journals.plos.org/plosone/s/editorial-and-peer-review-process#loc-peer-review-history)). If published, this will include your full peer review and any attached files.


If you choose “no”, your identity will remain anonymous but your review may still be made public.


**Do you want your identity to be public for this peer review?** For information about this choice, including consent withdrawal, please see our [Privacy Policy](https://www.plos.org/privacy-policy).

Reviewer #1: No

Reviewer #2: No

[NOTE: If reviewer comments were submitted as an attachment file, they will be attached to this email and accessible via the submission site. Please log into your account, locate the manuscript record, and check for the action link "View Attachments". If this link does not appear, there are no attachment files.]

While revising your submission, please upload your figure files to the Preflight Analysis and Conversion Engine (PACE) digital diagnostic tool, <https://pacev2.apexcovantage.com/>. PACE helps ensure that figures meet PLOS requirements. To use PACE, you must first register as a user. Registration is free. Then, login and navigate to the UPLOAD tab, where you will find detailed instructions on how to use the tool. If you encounter any issues or have any questions when using PACE, please email PLOS at [figures@plos.org](mailto:figures@plos.org). Please note that Supporting Information files do not need this step.
